# Dose optimization of cone beam computed tomography: measurement of parotid absorbed dose and image quality assessment

**DOI:** 10.25122/jml-2024-0168

**Published:** 2025-03

**Authors:** Lezan Othman Shina, Shereen Ismail Hajee

**Affiliations:** 1 Department of Dental Health, Hawler Specialized Center for Oral and Dental Health, Erbil, Kurdistan Region, Iraq; 2 Department of Pharmacology, Medical Physics and Clinical Biochemistry, College of Medicine, Hawler Medical University, Erbil, Kurdistan Region, Iraq

**Keywords:** cone-beam computed tomography, dental radiography, diagnostic imaging, radiation dosage, ALARA, As Low As Reasonably Achievable, CBCT, Cone-Beam Computed Tomography, CT, Computed Tomography, FOV, Field-Of-View, IQA, Image Quality Assessment, mA, milliamperage, TLD, ThermoLuminescent Dosimeters, 3D, Three-Dimensional, 2D, Two Dimensional

## Abstract

Cone-beam computed tomography (CBCT) is a three-dimensional (3D) imaging technology used in dentistry. This study aimed to reduce radiation exposure by adjusting CBCT parameters while ensuring that image quality remained suitable for diagnostic purposes. This controlled experimental study was conducted between February and July 2022 at the private Alpha Center for Dental Radiology in Erbil, Kurdistan Region of Iraq. The PaX-i3D SMART CBCT system, thermoluminescent dosimeters, and a specialized head and neck dosimetry phantom were used to measure the parotid gland. Tube voltage varied from 75 to 99 kVp, and tube current varied from 4 to 10 mA. For the image quality assessment, a dry human mandible immersed in water was exposed to CBCT X-rays with the same dosimetry exposure settings. Subjective image assessment was performed randomly by four dental and maxillofacial radiologists. The highest absorbed dose recorded was 654.47 µGy at 94 kVp and 8.1 mA, while the lowest was 198.5 µGy at 80 kVp and 4 mA. Out of the 32 scans, 19 images were considered acceptable based on clinical evaluation, and their absorbed dose ranges were lower than the default exposure setting of the device. Statistically, there was a strong positive correlation between absorbed dose, kVp, and mA, and a non-significant correlation between image quality and kVp in five (4, 4.5, 6, 8, and 10) of the seven mA groups. Optimizing CBCT settings to 80 kVp and 4 mA significantly reduced the radiation dose to the parotid gland while maintaining diagnostic image quality. This finding supports the adoption of lower mA and kVp settings in clinical practice to enhance patient safety without compromising diagnostic effectiveness.

## INTRODUCTION

The mouth and teeth are gateways to overall health, often requiring intricate diagnostic procedures to assess and treat dental and maxillofacial conditions [1]. Most dental imaging methods used in dental offices are two-dimensional (2D). These 2D projections have several drawbacks, including structural misrepresentation, superimposition, distortion, and magnification [2]. Computed tomography (CT) was the initial stage in the utilization of 3D projections in the fields of medicine and dentistry. The primary challenges hindering the wider adoption of CT in dentistry included its elevated expenses, limited availability, and the elevated levels of radiation exposure associated with its usage [3,4].

The advent of cone-beam computed tomography (CBCT) in 1998 by Mozzo *et al*. signified a significant paradigm shift in dentistry [5]. The pursuit of comprehensive visualization and documentation of the craniofacial complex has always been a fundamental objective in this field [4,6]. CBCT is a relatively new technology in dentistry and a result of dramatic advances in computer and electronic technology, along with similar advances in scanning and manufacturing [7]. It is becoming widely available and has applications in implant dentistry, endodontics, orthodontics, and pediatric and oral surgery. It has revolutionized maxillofacial imaging, expanding the role of imaging from diagnosis to guidance of operative and surgical procedures with the help of various software applications [8]. Although CBCT has a lower radiation exposure than conventional CT [3,9], concerns have been raised regarding the radiation dose implications of CBCT, which tend to be higher than those of conventional dental X-ray imaging methods such as panoramic and intraoral imaging techniques [10,11].

Radiological exposure involves the interaction of body tissues with ionizing radiation, which entails the risk of long-lasting cellular DNA modification and the development of latent tumors. The term 'stochastic effect' is employed to characterize an occurrence in which the level of risk is perceived to be higher for sensitive organs [12]. The brain, salivary glands, thyroid, and eye lens all experience elevated radiation levels owing to their positioning or proximity to the irradiated region in dental X-ray examinations, with the salivary glands absorbing notably higher doses [13]. During exposure, they are directly exposed to radiation and cannot be protected using shields, as they can be used for thyroid protection using a thyroid collar. Practitioners should be aware of the potential effects of ionizing radiation and understand the increased doses attributable to CBCT.

Optimization is defined as maintaining doses at levels that are 'as low as reasonably achievable' (ALARA) while ensuring that images are still of diagnostic quality [14]. Santos *et al*. showed that CBCT optimization protocols can significantly reduce the effective radiation dose while maintaining acceptable image quality by adjusting mA, projection images, and kVp [15]. The increasing utilization of CBCT in dentistry, with its higher radiation exposure than traditional 2D imaging, necessitates ongoing research to optimize radiation dose while ensuring diagnostic quality. Therefore, this study aimed to reduce the amount of radiation received by patients by adjusting the exposure settings while ensuring that the picture quality remained suitable for diagnostic purposes.

## MATERIAL AND METHODS

### Study design and setting

This controlled experimental study, conducted from February to July 2022 at the private Alpha Center for Dental Radiology in Erbil, Kurdistan Region of Iraq, examined the impact of different milliamperage and tube voltage settings on the radiation dose received by the parotid gland during CBCT scans. Dosimetry was assessed using thermoluminescent dosimeters (TLD), which were provided and analyzed by the central laboratories directorate in Baghdad. A customized head and neck phantom was created to measure the doses, while a dry human mandible phantom was utilized to evaluate the subjective image quality.

### CBCT machine

The Vatech Pax-i3D CBCT machine was used for this experiment. This device features a 360° rotating X-ray source, which completes a full revolution around the patient's head in 18 seconds, capturing 450 images during the scan. The Vatech Pax-i3D has user-controlled variables for the tube current (mA) and voltage (kVp), allowing precise adjustments. The commercially available options settings permit tube voltage variations from 60 to 99 kV in 1-kV increments and tube current variations from 4 to 10 mA in 0.1-mA increments. Additionally, the Vatech PaX-i3D CBCT machine allows the field of view (FOV) selection based on the region of interest, with three options: 5 × 5 cm, 10 × 7 cm, and 10 × 8.5 cm. The CBCT system was connected to a monitor running Ez3D-i software, and Lnk software was used for image processing.

### Dosimetry phantom

**Figure 1:**
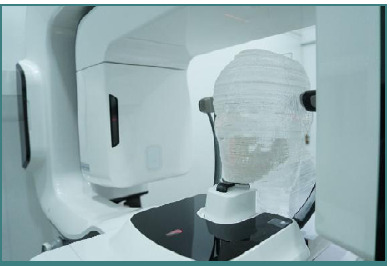
Dosimetry phantom

A special head and neck dosimetry phantom was used in this study to accurately simulate human tissue properties. The phantom was constructed from soft tissue-equivalent materials, with a urethane-based resin used to simulate the X-ray attenuation and density of human soft tissue. To replicate human bone characteristics, calcium carbonate (CaCO3) was combined with the urethane-based resin. Tissue-equivalent substitutes have physical properties similar to human tissue, such as density and attenuation coefficients, which are radiologically equivalent to soft tissue. The phantom was divided into 116 axial sections, each section width was approximately 2.5 mm. Anatomical structures, including specific organ locations, were carved based on an atlas of human anatomy, ensuring compatibility with the placement of thermoluminescent dosimeters on adjacent slices [7]. The phantom is shown in Figure 1.

**Figure 2:**
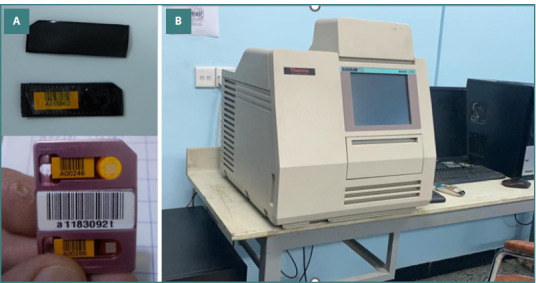
Thermoluminescent dosimeter (TLD) and reader setup for dose measurement. A, TLD encapsulated in a disposable vinyl pouch 7mm/cm^2^. B, TLD Reader, Harshaw Model 6600; Harshaw Thermo Fisher Scientific

### Dosimeter

All dose measurements were performed using TLD-100 (Li: Mg, Ti) dosimeters encapsulated in disposable vinyl pouches (7 mm/cm^2^) (Harshaw Thermo Fisher Scientific Inc.). These dosimeters are used for low-energy photon discrimination, enabling precise X-ray, beta, and neutron measurements [16]. All exposed TLDs were read using an automated TLD reader (Harshaw Model 6600; Harshaw Thermo Fisher Scientific, Figure 2AB).

### Procedure

The phantom was placed on a wooden stand and cardboard support to prevent scattering and to easily change its position. The alignment instruments of the machine, including a laser and chin rest, were used to position the phantom-like patient.

For this study, the largest FOV of 8.5 × 10 cm was selected to represent a mean adult head size. TLDs were placed on the carved side of the parotid gland. TLDs were placed using tweezers by the same operator. As previously indicated, the apparatus exhibits a kVp spectrum from 60 to 99 kVp. However, due to the limited number of TLDs available for assessment purposes, scans were conducted excluding TLDs to identify optimal scans and images. Those scans utilizing a kVp range below 70 kVp yielded unsatisfactory results, even with maximum mA settings. Consequently, they were omitted from the analysis. Additionally, exposure parameters exceeding the default settings of the equipment (8.1 mA and 94 kVp) were avoided to mitigate the absorbed radiation dose. Only one scan was included in the data analysis, which used 99 kVp; however, the current was the lowest at four mA. In total, 34 scans were performed at various tube voltage (70–90 kVp) and tube current (4–10 mA) settings. One scan auto-selected by the device (8.1 mA and 94 kVp) and another scan (60 kVp and 7 mA) were not included in the data analysis and were used to compare absorbed doses and image quality with the other 32 scan protocols. The remaining 32 scans were divided into seven exposure groups in each group; the tube current was fixed, but the tube voltages were different. The exposure settings are shown in Table 1. After exposure, the scanned TLDs were stored separately in individual bags and protected from light and radiation to be resent to the laboratory for reading, along with all exposed TLDs. A set of unexposed control TLDs was also sent back to obtain a background dose of 10, and the TLDs were used to measure the background signal. The results were reported as the organ-absorbed doses in the micrograys. The data were entered into a spreadsheet and analyzed for linearity, regression, and correlation.

**Table 1:** The seven groups of fixed mA with different kVp, with constant FOV of 8.5 x 10 cm

Group 1 (4 mA)		Group 2 (4.5 mA)		Group 3 (5.5 mA)		Group 4 (6 mA)		Group 5 (7.5 mA)		Group 6 (8 mA)		Group 7 (10 mA)	
No.	kVp	No.	kVp	No.	kVp	No.	kVp	No.	kVp	No.	kVp	No.	kVp
1	80	5	76	10	74	15	77	19	70	24	73	29	70
2	85	6	79	11	77	16	80	20	73	25	75	30	75
3	90	7	82	12	80	17	90	21	78	26	78	31	80
4	99	8	85	13	83	18	94	22	82	27	80	32	85
-	-	9	90	14	86	-	-	23	86	28	85	-	-

The selection of mA and kVp settings was based on a preliminary assessment of image quality and radiation dose, aiming to cover a broad range of clinically relevant exposure parameters. Each setting within the groups was chosen to represent a typical range of values used in clinical practice, ensuring a comprehensive analysis of their effects. Additionally, to ensure statistical validity, each exposure setting was repeated three times, and the mean values were used for analysis. This approach helped minimize variability and enhance the reliability of the results.

### Image quality assessment

**Figure 3:**
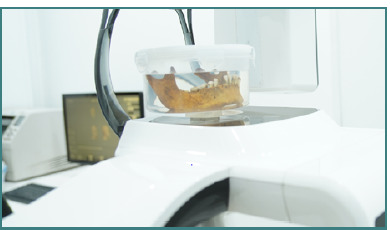
Dry mandible immersed in water for image quality assessment

For image quality assessment (IQA), a dry human mandible was obtained with approval from the College of Medicine (Hawler Medical University, Kurdistan region of Iraq). To simulate the soft tissue, a plastic container with a diameter of 20 cm filled with water was used. The mandible was immersed in the container and exposed to CBCT radiation during the scanning protocol, as shown in Figure 3.

Thirty-four scanning protocols were replicated using the identical machine specification and scanning protocol as the dosimetry segment. However, the dry mandible phantom was utilized instead of the dosimetry phantom and TLD. A questionnaire sheet was employed, incorporating six anatomical landmarks on the mandible. A three-point rating scale (0 = hardly visible, 1 = partially visible, 2 = well visible) was utilized to evaluate the visibility of the six anatomic structures and overall image noise.

**Rating 0:** The anatomical structure was scarcely discernible or entirely invisible. The image quality was substandard, with notable noise or artifacts hindering the structure. The boundaries of the structure were indistinct, complicating the identification and interpretation of anatomical details.

**Rating 1:** The anatomical structure was partly visible but lacked clarity. The image quality was fair, with some noise or artifacts present. Although the structure could be recognized, the details were not crisp, and certain parts might be obscured.

**Rating 2:** The anatomical structure was distinctly visible and well-defined. The image quality was excellent, with minimal noise or artifacts. The structure's edges were precise, and the details were easily distinguishable, facilitating precise identification and interpretation.

**Figure 4:**
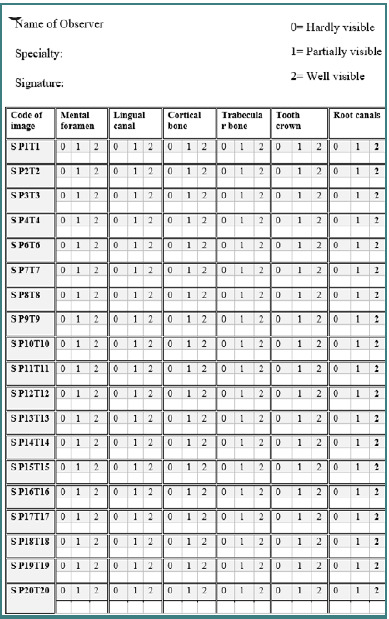
Image quality assessment sheet representing the selected anatomical landmarks of the mandible and the three-rating scales

Four observers assessed all images on the desktop with very good screen resolution and high performance (ASUS NB (AMD Ryzen 9 4900HS, Nvidia GeForce RTX 2060 Max-Q 6GB GDDR6, FHD 144 Hz IPS level,16GB DDR4-3200, 512GB PCIe). The observers were dentists, three were oral and maxillofacial radiologists, and one was an oral surgeon. All observers were trained using the software tool used in this study before conducting observations. The observers were blinded to the selected scan protocols and could manipulate the screen settings, such as sharpness and screen layout, questionnaire sheet, anatomical landmarks, and rating scales, as shown in Figure 4.

### Statistical analysis

The statistical analysis was conducted using IBM SPSS version 28 and GraphPad Prism version 8. Descriptive statistics were used to summarize the data. Correlation analyses were conducted to explore the relationships between independent and dependent variables. Pearson correlation coefficients were calculated to assess the strength and direction of these relationships. Scatter plots were generated to visually represent the correlations.

## Results

### Clinical analysis

The most elevated absorbed dose recorded was 654.47 µGy, representing the default exposure configuration of the device, whereas the least absorbed dose noted was 120.49 µGy, denoting the minimum exposure setting (Table 2).

**Table 2:** The absorbed dose and image quality level of the auto-selected and minimum exposure settings of the device

KVp	mA	Absorbed dose µGy	Image quality level
60	7	120.49	Fair
94	8.1	654.47	very good

Table 3 presents a comprehensive analysis of absorbed doses and image quality levels across seven mA groups with varying kVp settings. The absorbed doses ranged from 140.98 µGy to 603.26 µGy, with image quality levels classified as very good, good, or fair. Specifically, the mA 4 group demonstrated absorbed doses between 198.5 µGy and 323.98 µGy, with three scans rated as very good and one as good. The mA 4.5 group exhibited doses from 140.98 µGy to 222.72 µGy, with two scans rated as very good, one as good, and two as fair. The mA 5.5 group showed doses from 148.7 µGy to 281.78 µGy, with three scans rated as very good, one good, and two as fair. The mA 6 group had doses ranging from 241.76 µGy to 603.26 µGy, with four scans rated as very good and one as good. The mA 7.5 group displayed doses from 238.96 µGy to 337.41 µGy, with three scans rated as very good, one good, and two as fair. The mA 8 group showed doses from 307.48 µGy to 590.2 µGy, with five scans rated as very good and one as good. Lastly, the mA 10 group exhibited doses from 326.76 µGy to 574.8 µGy, with four scans rated as very good and one as fair. Notably, the optimal protocol for dose optimization was identified as scan number one (80 kVp, 4 mA) with the lowest absorbed dose of 198.5 µGy and a very good image quality rating.

**Table 3:** The absorbed dose and image quality level across all seven mA groups

mA groups	No.	KVp	Absorbed dose µGy	Image quality level
mA 4	1	80	198.5	very good
	2	85	208.21	very good
	3	90	244.96	very good
	4	99	323.98	Good
mA 4.5	5	76	140.98	Fair
	6	79	181.16	Fair
	7	82	190.95	Good
	8	85	222.72	very good
	9	90	211.31	very good
mA 5.5	10	74	148.7	Fair
	11	77	169.98	Fair
	12	80	198.17	Good
	13	83	269.73	very good
	14	86	281.78	very good
mA 6	15	77	241.76	very good
	16	80	273.15	Good
	17	90	327.87	very good
	18	94	603.26	very good
mA 7.5	19	70	238.96	Fair
	20	73	272.21	Fair
	21	78	294.97	Good
	22	82	324.97	very good
	23	86	337.41	very good
mA 8	24	73	307.48	Good
	25	75	359.73	very good
	26	78	345.23	very good
	27	80	369.93	very good
	28	85	590.2	very good
mA 10	29	70	326.76	Fair
	30	75	338.11	very good
	31	80	445.46	very good
	32	85	574.8	very good

**Figure 5:**
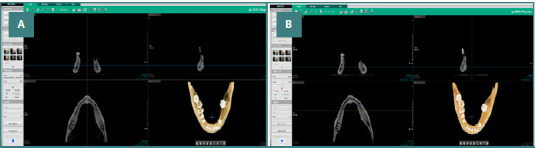
Effect of exposure settings on absorbed dose and image quality in CBCT imaging. A, Image obtained using the default exposure of the device (kVp: 94, mA: 8.1), resulting in an absorbed dose of 654.47 µGy with an image quality (IQ) level of 'very good'. B, Image obtained using the manually selected exposure setting (kVp: 80, mA: 4, absorbed dose: 198.5 µGy), which resulted in the lowest absorbed dose among all scan protocols while maintaining a very good IQ level.

Amongst the 19 scans assessed as of exceptional quality, it is worth noting that a particular scan protocol (80 kVp, 4 mA) stood out for delivering the minimum absorbed dose of approximately 198.5 µGy, as depicted in Figure 5AB.

### Correlation and regression

#### Correlation between absorbed dose and kVp

**Figure 6:**
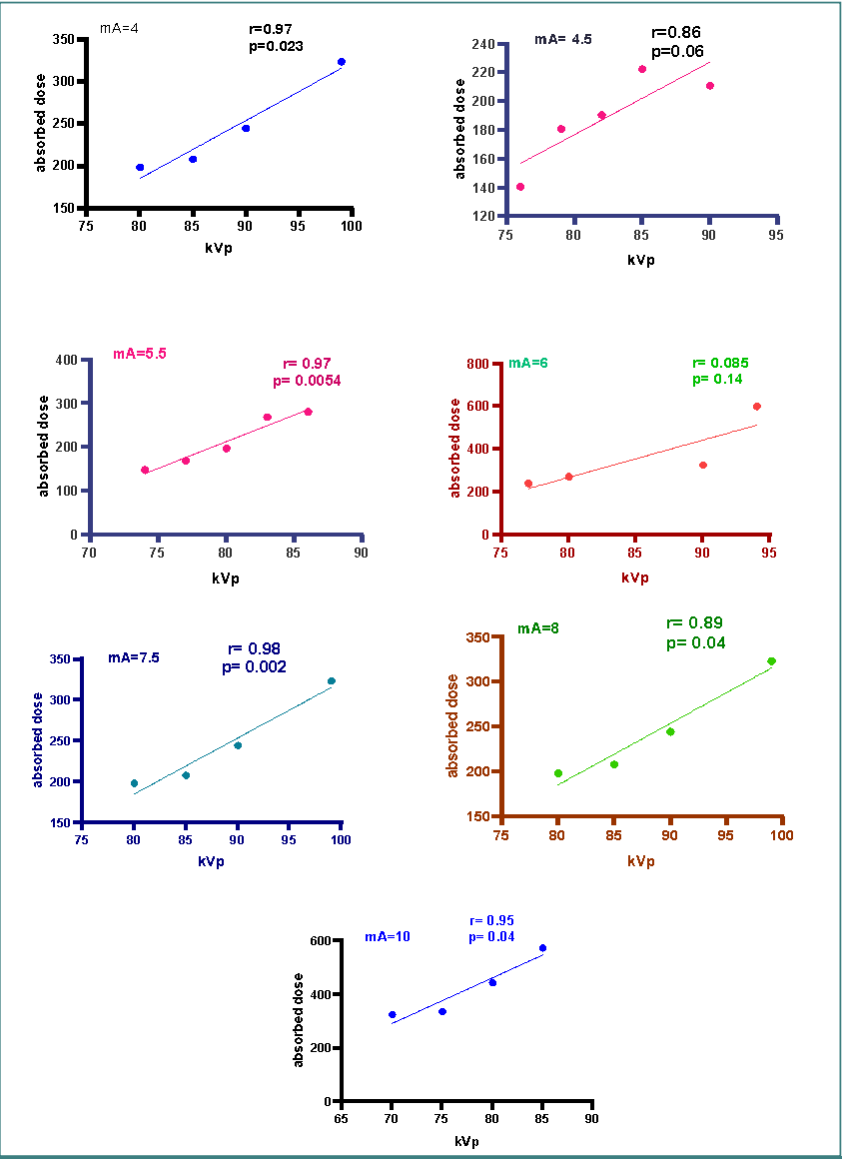
Seven scatter dot graphs show the significant correlation between parotid absorbed dose and kVp with seven different mA (4, 4.5, 5.5, 6, 7.5, 8, and 10) groups

As shown in Figure 6, there was a statistically significant and very strong positive correlation between each tube voltage and the parotid absorbed dose within specific tube current groups. The correlation coefficients for 4 mA (r = 0.976), 5.5 mA (r = 0.973*), 7.5 mA (r = 0.985**), 8 mA (r = 0.898*), and 10 mA (r = 0.952*)** indicate a strong linear relationship, as their *P* values were below the significance thresholds of α = 0.01 and α = 0.05. In contrast, no statistically significant strong correlation was observed between tube voltage and parotid absorbed dose in the 4.5 mA (r = 0.862, *P* > 0.05), 6 mA (r = 0.854, *P* > 0.05), and combined tube current groups (all mA settings together: r = 0.303, *P* > 0.05).

#### Correlation between tube voltage and image quality in the seven groups of tube currents

**Figure 7:**
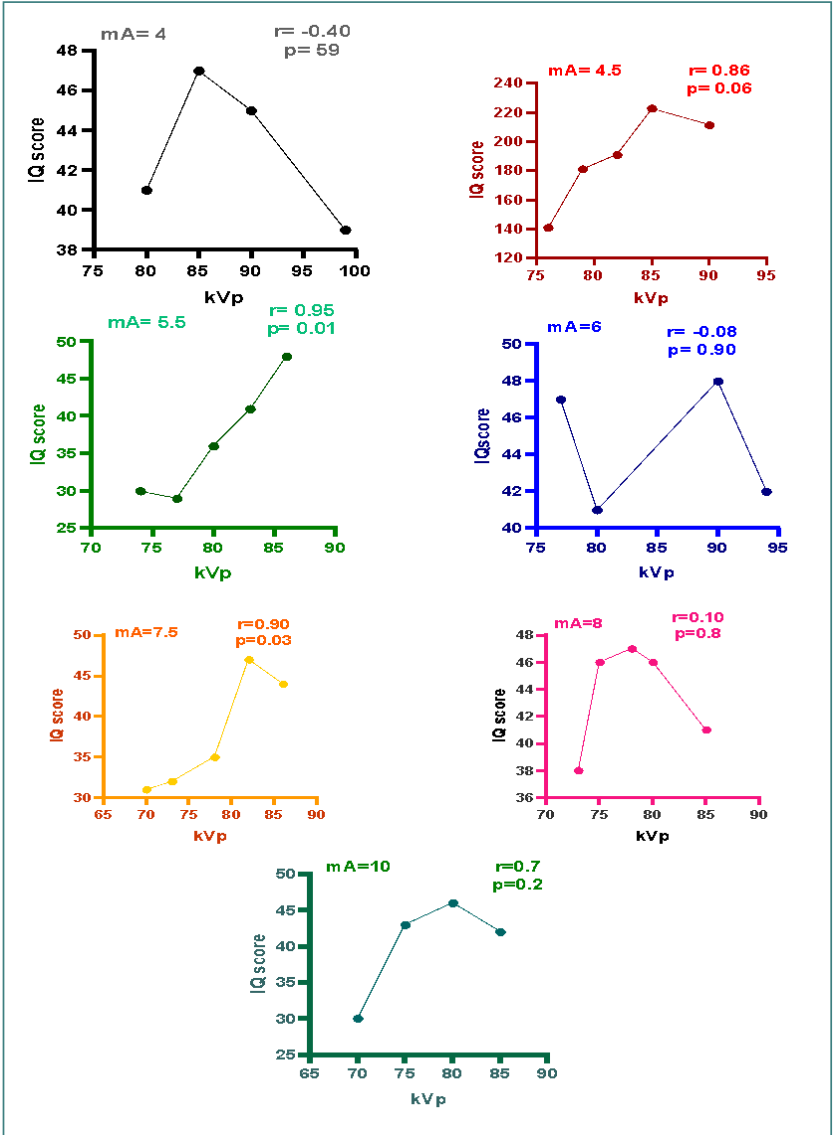
Seven scatter dot graphs show the significant correlation between kVp and image quality with seven different mA (4, 4.5, 5.5, 6, 7.5, 8, and 10) groups

As illustrated in Figure 7, there was a statistically significant and very strong positive correlation observed for both 5.5 mA (r = 0.958, *P* < 0.05) and 7.5 mA (r = 0.952, *P* < 0.05). Consequently, these settings also showed a strong correlation with the overall observer scores for image quality, as their *P* values were below the significance threshold (α = 0.05).

In contrast, no statistically significant correlation was found for 4 mA (r = -0.406, *P* > 0.05), 4.5 mA (r = 0.841, *P* > 0.05), 6 mA (r = 0.073, *P* > 0.05), 8 mA (r = 0.102, *P* > 0.05), and 10 mA (r = 0.715, *P* > 0.05). Consequently, images obtained within this mA range (4, 4.5, 6, 8, and 10 mA) demonstrated image quality within the diagnostic range despite the lack of a statistically significant correlation.

*. Correlation is significant at the 0.05 level (2-tailed).

**. Correlation is significant at the 0.01 level (2-tailed).

## Discussion

The present study aimed to optimize the absorbed dose in radiographic imaging while maintaining high image quality, a critical balance in clinical diagnostics. In the present study, the highest absorbed dose recorded was 654.47 µGy at the default setting of the device, while the lowest was 120.49 µGy at the minimum setting. Absorbed doses ranged from 140.98 µGy to 603.26 µGy across the seven mA groups, with image quality rated as very good, good, or fair. The optimal protocol identified was 80 kVp and 4 mA, yielding the lowest absorbed dose of 198.5 µGy with very good image quality. Strong positive correlations were found between tube voltage and absorbed dose in most mA groups.

In 2012, the European Commission published Radiation Protection No. 172, an evidence-based guide for the safe use of CBCT in dental radiology. This guide is part of the SEDENTEXCT project, which aims to acquire the main scientific information on the clinical use of CBCT, in addition to presenting the basic principles of radiological protection, such as justification, optimization of exposures, user training, and quality assurance. The use of CBCT in dentistry should only be justified if it provides new information that cannot be obtained using panoramic radiography or another technique with a lower radiation dose [17-19].

The present study results indicate that the absorbed dose varies significantly across different mA and kVp settings, with the lowest absorbed dose recorded at 198.5 µGy for the 80 kVp, 4 mA setting, which also maintained a very good image quality rating. This finding aligns with previous research emphasizing the potential for dose optimization through careful adjustment of exposure parameters [20]. The study by Al-Saeed *et al*. highlighted that lower mA settings, when paired with appropriate kVp, can significantly reduce patient dose while preserving image quality, a principle corroborated by the present study data [21].

The correlation analysis revealed a statistically significant and strong positive correlation between tube voltage and absorbed dose in several mA groups, particularly at 4 mA, 5.5 mA, 7.5 mA, 8 mA, and 10 mA. This suggests that higher kVp settings inherently increase the absorbed dose, a finding consistent with the literature. For instance, a study by Jha *et al*. aimed at estimating the projected cancer risk from CBCT for orthodontic purposes found that higher exposure settings (e.g., 130 kVp/200 mAs) resulted in higher organ doses compared to median exposure settings (e.g., 105 kVp/156.8 mAs) [22]. However, the absence of a significant correlation in the 4.5 mA and 6 mA groups indicates that other factors, such as patient anatomy and specific imaging protocols, may influence dose absorption.

The study also examined the relationship between kVp and image quality across different mA settings. According to the results of the present study, the absorbed dose ranged from 654.47 Gy to 198.5 Gy. Decreasing kVp had a greater impact on the absorbed dose than decreasing mA; even with low kVp, the absorbed dose in the current group of 6 mA was higher than that in the scans using 8 mA. On the other hand, regarding tube voltage optimization, a higher kVp is typically thought to provide better image quality. However, this study demonstrated a significant reduction in the absorbed dose when the device exposure parameters were changed from 10 mA to 4 mA and 90 kVp to 70 kVp under typical conditions, even with a large FOV. The findings of the two further studies by Pauwels *et al*. and Oenning *et al*. support this. Low-exposure settings were used in both experiments to significantly reduce the dosage [23,24].

In this study, the absorbed dose accrued within the region corresponding to the parotid gland exhibited the highest magnitude under the default exposure parameters of the equipment, characterized by a voltage of 94 kVp and a current intensity of 8.1 mA, while ensuring optimal image quality. Fortunately, upon juxtaposing the default exposure image with images generated using alternative lower scan protocols, it is evident that high-quality images can still be produced. For instance, in the initial scan protocol, reducing the voltage to 80 kVp and the current intensity to 4 mA resulted in an image that can rival the default scan in terms of quality while concurrently reducing the dose from 654.47 µGy to 198.5 µGy. Therefore, the implications of these findings for clinical practice are profound. By identifying the optimal exposure settings that minimize absorbed dose while maintaining high image quality, this study contributes to the ongoing efforts to enhance patient safety in radiographic imaging. The optimal protocol identified (80 kVp, 4 mA) offers a practical guideline for clinicians aiming to reduce radiation exposure without sacrificing diagnostic accuracy. This aligns with the principles of the ALARA framework, which advocates for minimizing radiation exposure in medical imaging (ICRP, 2007) [14].

### Strengths and limitations

One of the major strengths of this study lies in its comprehensive data collection across a range of mA and kVp settings, providing a robust dataset for analysis. Using a systematic approach to evaluate the correlation between absorbed doses and image quality further strengthens the conclusions' reliability. However, the present study had several limitations. Only one phantom was used, which could affect the generalizability and accuracy of the dose measurements and image quality assessments. Additionally, the image quality assessment relied on subjective ratings by four observers, which may introduce observer bias and variability. Finally, the study's findings are based on a controlled experimental setting, which may not fully replicate clinical conditions.

## CONCLUSION

In conclusion, this study successfully identified optimal exposure settings for radiographic imaging that minimize absorbed dose while maintaining high image quality. Specifically, the optimal protocol of 80 kVp and 4 mA significantly reduced absorbed doses to as low as 198.5 µGy, demonstrating very good image quality. This finding underscores the potential for significant reductions in patient radiation exposure through careful adjustment of imaging parameters, which is in line with the ALARA principle of minimizing radiation as much as reasonably achievable.

## Personal thanks

The authors thank all staff at the Alpha Center for Dental and Maxillofacial Radiology in Erbil, Kurdistan region of Iraq, for their help with data collection.
